# Editorial: Research advances of modification and nutrition properties of food carbohydrates, volume I

**DOI:** 10.3389/fnut.2023.1270049

**Published:** 2023-10-04

**Authors:** Yanjun Zhang, Jianhua Xie, Bin Li, Srinivas Janaswamy

**Affiliations:** ^1^Spice and Beverage Research Institute, Chinese Academy of Tropical Agricultural Sciences, Wanning, China; ^2^Key Laboratory of Processing Suitability and Quality Control of the Special Tropical Crops of Hainan Province, Wanning, Hainan, China; ^3^Department of Food Nutrition, State Key Laboratory of Food Science and Resources, Nanchang University, Nanchang, China; ^4^Department of Food Processing, College of Food Science and Engineering, Shenyang Agricultural University, Shenyang, China; ^5^Department of Dairy and Food Science, South Dakota State University, Brookings, SD, United States

**Keywords:** starch, non-starch polysaccharides, dietary fiber, cellulose, hemicellulose

## 1. Introduction

As a group of essential biopolymers, carbohydrates exist widely in living organisms and play many known and unknown biological roles in life activities via different pathways. Carbohydrates are widely used in foodstuffs, pharmaceuticals, biofuels, and biomaterials, to name a few. Parallelly, a growing understanding and deeper investigation drive the development of natural carbohydrates for novel applications, especially for treating chronic diseases, e.g., hyperlipidemia, obesity, and diabetes. The emerging evidence indicates that carbohydrates are effective for modulating gut microbiota, a vital organ in health and diseases. In addition, modifying carbohydrates alters and/or enhances nutrition properties, further expanding their application potential. Notably, the nutritional properties of carbohydrates depend on their chemical structures and chain conformations. Thus, structural identification of carbohydrates and their derivatives helps expand their food, pharmaceutical, and related applications. To this end, a Research Topic entitled “*Research Advances of Modification and Nutrition Properties of Food Carbohydrates*” was launched by Frontiers in Nutrition, Food Chemistry (Frontiers) to provide a forum for researchers to disseminate their latest research findings on starch, non-starch polysaccharides, dietary fiber, pectin, cellulose, hemicellulose, and other food components. A total of 16 manuscripts from various countries were submitted, of which 12 were accepted for publication after the peer review, including two reviews and 10 research articles.

## 2. Starch

There were many studies on the modification and nutritional properties of food carbohydrates. In this set, starch is widely used in the food industry for bakery items, noodles, instant foods, and snacks. However, poor solubility, instability during pasting, undesired consistency, and retrogradation of native starches limit their widespread utility. To address these issues, physical, chemical, and enzymatic modifications and their combinations have been employed to enhance the physicochemical properties of starch. These modifications improve solubility, stability, and consistency and reduce retrogradation, making starch more suitable for various food applications. Gao et al. isolated and purified Lanzhou Lily (*Lilium davidii* var. unicolor) by polyethylene glycol-based ultrasonic-assisted enzymatic extraction method (PEG-UAEE). Single-factor experiments and response surface methodology (RSM) established the most effective process conditions. Subsequently, the preliminary structure of the low-molecular-weight polysaccharides (LLPs) was characterized using HPLC, FT-IR, and SEM, and their antioxidant activities were assessed. The findings demonstrated that the optimized conditions resulted in an LLPs yield of 14.75%: enzyme-to-substrate (E/S) ratio of 1,400 U/g; pH of 5.0; ultrasonic time of 30 min; and ultrasonic temperature of 50°C. The LLPs exhibited a pyranose ring, uronic acid, and characteristic absorption peaks of –OH, C=O, and C–H. Scanning electron microscopy revealed irregular distribution, dispersed structure, and numerous pores in the LLPs. HPLC analysis indicated that the LLPs were heteropolysaccharides consisting of galactose (6.36%), glucose (76.03%), rhamnose (2.02%), and arabinose (7.09%). Furthermore, *in vitro* testing demonstrated significant antioxidant effects of the LLPs. These results suggest that LLPs hold potential for applications as natural antioxidants and functional food ingredients. Yan et al. provided an overview of the production strategies employed for xylooligosaccharides (XOS) and discussed the raw materials, preparation methods, and purification techniques. Additionally, they presented the biological characteristics and applications of XOS. The most commonly recommended approach for XOS production is the two-stage method involving alkaline pre-treatment and enzymatic hydrolysis, with subsequent membrane filtration for enhanced yield and prebiotic functionality. Furthermore, novel strategies and technologies such as hydrothermal and steam explosion methods have also been explored, combined with enzymatic hydrolysis to produce XOS. It exhibits various significant physiological activities, particularly regulating blood glucose, reducing blood lipid levels, and improving the host intestinal flora structure. Furthermore, an additional study examined the correlation between glycemic release characteristics and the fine supramolecular structure of starches derived from cassava (ECS), potato (EPS), jackfruit seed (EJFSS), maize (EMS), wheat (EWS), and rice (ERS). These starches were prepared using an improved extrusion modification technology (IEMS). The results revealed that the extruded cooking starches transitioned from the A-type to V-type crystal structure. Specifically, IEMS-treated cassava, potato, and rice starches displayed broken α-1,6-glycosidic amylopectin (long chains), while the others exhibited sheared α-1,4-glycosidic amylopectin. The molecular weight, medium and long chain counts, and relative crystallinity decreased while the number of amylopectin short chains increased. ECS, EPS, EJFSS, and EWS demonstrated improved glycemic index (GI) and digestive speed rate constant (k) compared to raw starch. Although EMS and ERS exhibited degraded molecular structures, their particle morphology transformed from looser polyhedral shapes to more compact ones with fewer enzymolysis channels due to the rearrangement of side chain clusters of amylopectin, resulting in enhanced enzyme resistance. Notably, the IEMS-treated samples exhibited significant variations in starch characteristics. EPS had the highest amylose content, medium chains, long chains, and molecular weight but with the lowest GI, relative crystallinity, and k. Conversely, ERS displayed an opposite trend. Consequently, IEMS impacts starches with variable GIs. This investigation provides a foundation for broader applications of conventional crop starches in the food industry catering to diverse nutritional needs (Li, Zhang, et al.). Li L. et al. investigated the structural properties and physicochemical characteristics of lotus seed cross-linked resistant starches (LSCSs). Various concentrations of crosslinking agents were used to produce eight samples LS-0CS, LS-1CS, LS-2CS, LS-4CS, LS-6CS, LS-8CS, LS-10CS, and LS-12CS. The degree of crosslinking increased with higher crosslinking, leading to greater LSCS granular agglomeration. As observed in FT-IR analysis the P=O vibration at 1,250 cm^−1^ confirmed the crosslinking reaction. The covalent bonds formed by the phosphate groups were primarily composed of distarch monophosphate (DMSP), as determined by ^31^P NMR. As the degree of crosslinking increased, the peak strength of DMSP became stronger, and the specific gravity increased. Among the eight samples, LS-12CS exhibited the highest degree of crosslinking and the greatest specific gravity. Additionally, the solubility of LSCSs decreased, while thermal stability and resistance to digestion improved with increasing crosslinking, which correlated with the degree of agglomeration and the presence of DMSP. LS-12CS displayed a resistant starch (RS) content of 48.95 ± 0.86%. With its low solubility, heat resistance, and high RS content, LS-12CS demonstrates potential as a prebiotic ingredient for the food industry. Zeng et al. summarized the effects of various dietary compounds, including cell walls, proteins, lipids, non-starchy polysaccharides, and polyphenols, on the enzymatic digestion of starch. These compounds were found to have distinct impacts on the digestion process. Cell walls, proteins, and non-starchy polysaccharides hindered starch disruption during hydrothermal treatment, preserving ordered structures that limited enzymatic binding. Additionally, these compounds encapsulated starch granules and acted as physical barriers, preventing enzymes from accessing the starch. Proteins, non-starchy polysaccharides, lipids, and polyphenols interacted with starch and formed organized assemblies. Furthermore, non-starchy polysaccharides and polyphenols exhibited a strong ability to reduce the activities of α-amylase and α-glucosidase. Based on these findings, it can be concluded that dietary compounds play a role in reducing starch digestion through three main mechanisms: (i) preservation of ordered structures and formation of organized assemblies with dietary compounds; (ii) creation of physical barriers that prevent enzyme access and binding to starch; and (iii) inhibition of enzyme activities. Modulating starch enzymatic digestion by dietary compounds holds significant potential in regulating postprandial glucose response to food and preventing or treating type II diabetes. Li, Xie, et al. isolated starches from Chinese mutant *Musa acuminata* Colla acuminata and double balbisiana (MA), Musa double acuminata cv. Pisang Mas (MAM), *Musa acuminata* cv. Pisang Awak (MAA), Musa Basjoo Siebold (MBS), Musa double acuminata and balbisiana-Prata (MAP). The results showed that all the starches had a high amylose content, ranging from 34.04 to 42.59%. Based on particle size, the starches were categorized into two groups: medium-sized (MA, MAM) with particle sizes ranging from 14.54 to 17.71 μm, and large-sizes (MAA, MBS, and MAP) from 23.01 to 23.82 μm. The medium-sized starches exhibited A-type crystallization, higher peak viscosity, final viscosity, gel fracturability, and gel hardness. On the other hand, the large-sized starches presented B-type crystallization, compact particle morphology, a higher degree of crystallinity, short-range order, gelatinization enthalpy, pasting temperature, lower porosity, and higher water absorption capacity (WAC), and oil absorption capacity. The medium-sized starches with higher peak viscosity and gel hardness were found suitable as food thickening or gelling agents. The large-sized starches, with their unique characteristics such as higher degree of crystallinity, lower porosity, and WAC, showed potential as materials for resistant starch production. The study highlighted the significant influence of amylose content on the microstructure and physicochemical properties of starch samples. Overall, these findings suggested that the amylose content plays a crucial role in determining the microstructure and properties of starches, and provide opportunities for utilization in various food applications.

## 3. Non-starch polysaccharides

Non-starch polysaccharides find extensive application in various foods, including candies, pastries, and dairy products. They offer nutritional benefits and enhance the quality and texture of these foods through gelation, water holding, binding, foaming, stability, solubility, emulsification, and other properties. However, functional properties often fall short of expectations. Several methods, such as physical, chemical, and enzymatic modifications, are handy to improve the functional properties. He et al. summarized an overview of the chemical structures and probiotic potential of polysaccharides (LPs) extracted from fermented litchi pulp using *Lactobacillus fermentum* for different durations (ranging from 0 to 72 h, corresponding to LP-0 through LP-72, respectively). The fermentation time impacted the yields, total sugar content, uronic acid content, molecular weight, and monosaccharide composition of LPs. The LPs' yields and uronic acid content displayed irregular trends with fermentation time, while the total sugar content decreased and the molecular weight increased. Notably, LP-6 exhibited the highest extraction yield (2.67%), lowest uronic acid content, and smallest average molecular weight (104 kDa; *p* < 0.05). Analysis of the monosaccharide composition in the fermented LPs indicated decreased glucose proportions, whereas arabinose and galacturonic acid proportions increased compared to unfermented LP-0. Furthermore, LP-6 demonstrated the highest growth stimulation for Bifidobacterium compared to LP-0, while other fermentation durations exhibited comparable or inferior probiotic-promoting activities. These findings suggest that fermentation by lactic acid bacteria alters the physicochemical properties of litchi polysaccharides, and selecting an appropriate fermentation duration can enhance their probiotic activities. They further indicate that a proper fermentation time by lactic acid bacteria for litchi pulp might facilitate the probiotic properties of its polysaccharides. Wei et al. evaluated the relationship between the characteristics of regional rice as raw material and the resulting quality of rice noodles. Four commonly used rice cultivars for noodle production in Guangxi were examined. The findings revealed that the composition of rice flour significantly influenced gelatinization and retrogradation, which in turn affected the textural and sensory properties of rice noodles. The amylose content exhibited a strong positive correlation with the peak viscosity (PV) and trough viscosity (TV) of rice flour (*p* < 0.01). PV and TV showed strong negative correlations with adhesive strength (*p* < 0.01) and positive correlations with chewiness (*p* < 0.05), hardness, peak load, and deformation at the peak of rice noodles (*p* < 0.01). The protein content demonstrated a positive correlation with the setback of rice flour (*p* < 0.05), which is known to influence retrogradation. Additionally, solubility exhibited positive correlations with cooking loss (*p* < 0.01) and broken rate (*p* < 0.05) of rice noodles and a strong negative correlation with springiness (*p* < 0.01). Swelling power negatively correlated with the broken rate (*p* < 0.05). As the sensory score of rice noodles was negatively correlated with the broken rate (*p* < 0.05) and cooking loss (*p* < 0.01) and positively correlated with springiness (*p* < 0.01), it can be inferred that the solubility and swelling power of rice flours are useful indicators for predicting consumer acceptability of rice noodles. Ji et al. isolated a new polysaccharide (PZMP3-1) from *Ziziphus Jujuba* cv. Muzao fruit, and composition, molecular weight, and principal structural components were examined. It contains 2.56 rhamnose, 7.70 arabinose, 3.73 galactose, and 6.73 galactose, with an average molecular weight of 241 kDa. Methylation and nuclear magnetic resonance spectroscopy (NMR) analyses identified the key structural components, including 1,2,4 and 1,4-linked GalpA, 1,4-linked Galp, 1,3 and 1,5-linked Araf, and 1-linked Rhap. Structural analysis using X-ray diffraction (XRD), Fourier transform infrared spectroscopy (FT-IR), atomic force microscopy (AFM), and scanning electron microscopy (SEM) revealed a tangled and branching pattern. Overall, PZMP3-1 possesses unique bioactivities and potential for wide applications in nutritional supplements. Yang et al. conducted an extraction of polysaccharides from *Sibiraea laexigata* (L.) Maxim (SLM) and purified two fractions of SLM polysaccharides (SLMPs) named SLMPs-1-1 and SLMPs-2-1 using DEAE Cellulose-52 and Sephadex G-100 chromatography. The preliminary structure of these two fractions was established through various analyses such as chemical composition, molecular weight measurement, UVS, HPLC-PMP, FTIR, nuclear magnetic resonance (NMR) spectroscopy, and SEM. The results revealed that the two fractions had different molecular weights of 1.03 and 1.02 kDa composed of glucose (46.76 and 46.79%, respectively). The structural characterization using FT-IR, 1H NMR, and SEM indicated that SLMPs-1-1 and SLMPs-2-1 exhibited typical pyranose polysaccharide characteristics with α-glycosidic and β-glycosidic bonds. Additionally, it was observed that SLMPs-1-1 could increase the levels of tumor necrosis factor-alpha (TNF-α) and interleukin-2 (IL-2) while mitigating immune organ tissue damage in cyclophosphamide (Cy)-treated mice. The results from RT-qPCR and Western blot analysis showed that SLMPs-1-1 significantly upregulated the levels of NF-kB and TLR4, indicating its potential involvement in the immunosuppressive protection of Cy-treated mice. These findings suggest that SLMPs-1-1 has the potential to serve as an alternative immunostimulatory and could find applications in the food and pharmaceutical industries. Zeng et al. examined the effects of polysaccharides from *Artocarpus heterophyllus* Lam. pulp (jackfruit, JFP-Ps) on intestinal barrier function. The researchers investigated the impact of JFP-Ps on intestinal health by performing H&E staining and biochemical analysis to assess the pathological and inflammatory state of the intestine, as well as oxidative damage. They also analyzed the expression of genes and proteins associated with intestinal health and inflammation using RT-qPCR and western blotting. The results demonstrated that JFP-Ps had several beneficial effects on intestinal barrier function. Firstly, JFP-Ps promoted bowel movement and modified the physiochemical environment of the intestine by reducing fecal pH and increasing fecal water content. Additionally, they alleviated oxidative damage in the colon, relieved intestinal colonic inflammation, and regulated blood glucose transport in the small intestine. They further repaired intestinal mucosal damage, increased the thickness of the mucus layer, and improved intestinal physiological status. Furthermore, they downregulated the expression of inflammatory genes such as TNF-α and IL-6, while upregulating the expression of free fatty acid receptors (GPR41 and GPR43) and tight junction protein (occludin). These findings suggest that JFP-Ps exert a protective effect on intestinal function by enhancing the intestine's biological, mucosal, immune, and mechanical barrier functions. JFP-Ps also activate signaling pathways related to short-chain fatty acids (SCFAs) and GPR41/GPR43. Based on these results, JFP-Ps show promise as a natural compound for improving human intestinal health and may be used as a potential phytochemical for this purpose. Gao et al. (1) extracted polysaccharides (ALPs) from *Arctium lappa* L. using an optimized aqueous two-phase system with specific conditions: polyethylene glycol (PEG) relative molecular weight of 6,000, PEG quality fraction of 25%, (NH_4_)_2_SO_4_ quality fraction of 18%, and extraction temperature of 80°C. The extraction rate reached 28.83%. FTIR, SEM, and HPLC analyses revealed that ALPs were acidic heteropolysaccharides with uneven particle size distribution, irregular shape, and rough surface. The composition of ALPs consisted primarily of glucose, rhamnose, arabinose, and galactose, with molar ratios of 70.19:10.95:11.16:6.90, respectively. Additionally, ALPs exhibited strong *in vitro* antioxidant activity, effectively scavenging hydroxyl radicals (^·^OH), DPPH radicals, and superoxide anions, with IC50 values of 1.732 mg/ml, 0.29 mg/ml, and 0.15 mglmL, respectively. These findings highlight the potent antioxidant properties of ALPs and their potential as functional food ingredients.

## 4. Dietary fiber, cellulose and hemicellulose

Dietary fiber, a non-digestible polysaccharide, is considered a crucial nutrient by the nutritional community. It cannot be absorbed by the gastrointestinal tract, making it unique among nutrients. Enhancing insoluble dietary fiber's quality and functional properties through physical, chemical, and biological methods is essential for its extensive use in the food industry. These approaches aim to improve its characteristics and make it more suitable for various food applications. Wang et al. utilized tigernut to synthesize soluble dietary fiber-manganize complex [SDF-Mn(II)]. Comprehensive microscopic and structural analyses were conducted, including scanning electron microscopy, Fourier infrared spectroscopy, UV full-band scanning, X-ray diffraction, thermal analysis, gel permeation chromatography, and nuclear magnetic resonance. The *in vitro* hypoglycemic activity of SDF-Mn(II) was also investigated. The results revealed that the interaction between Mn(II) and SDF primarily involved hydroxyl and carbonyl groups. Nuclear magnetic resonance analysis demonstrated specific covalent bonding and substitution primarily occurring at the C6 position. Compared to SDF alone, the SDF-Mn(II) complex exhibited a porous structure, induced a red-shift and enhanced color intensity in UV characteristic peaks, displayed increased crystallinity, reduced molecular weight, and improved thermal stability. Furthermore, SDF-Mn(II) demonstrated significantly enhanced inhibition of α-amylase and α-glucosidase, indicating potent *in vitro* digestive enzyme inhibition activity.

Cellulose and hemicellulose, like non-starch polysaccharides, are widely utilized in various food applications. They contribute not only to the nutritional value but also to the quality and texture of foods due to their gelation, water retention, binding, foaming, stability, solubility, emulsification, and other functional properties. However, the inherent functional limitations of cellulose and hemicellulose often necessitate improvements. Various methods, including physical, chemical, and enzymatic modifications, have been employed to enhance the functional properties of cellulose and hemicellulose, allowing for their broader utilization in the food industry. Su et al. (2) found that cellulose and hemicellulose edible films with 10% microcapsule content showed the best overall performance, improving mechanical properties, thermal stability, and barrier properties. The films effectively inhibited *Listeria monocytogenes* (93.69%) and *Escherichia coli* (95.55%) and suppressed the growth of *Staphylococcus griseus*. When used for blueberry preservation, the ClO_2_ self-releasing films delayed quality decline and prevented mold contamination during a 14-day storage period. Additionally, the antibacterial film group exhibited higher anthocyanin accumulation. These findings suggest that films containing ClO_2_ microcapsules hold promise for future fruit and vegetable packaging.

## 5. Conclusions

The articles in this Research Topic encompass a wide range of topics, including enhancing the physical properties of key food components, investigations into biological activities, and applications in food preservation. Pursuing desirable food texture and taste, as well as the increasing demand for safe, environmentally friendly, and efficient food materials, are the main motivations driving ongoing research in this field. We hope these novel research findings find practical and more useful applications in food and related applications.

## Author contributions

YZ: Writing—original draft, Writing—review and editing. JX: Writing—review and editing. BL: Writing—review and editing. SJ: Writing—review and editing.
